# The Effects of Overweight and Obesity on Obstacle Crossing During Walking: Protocol for a Systematic Review

**DOI:** 10.2196/36234

**Published:** 2022-05-20

**Authors:** Matthias Chardon, Fabio Augusto Barbieri, Tiago Penedo, Paulo Cezar Rocha Santos, Nicolas Vuillerme

**Affiliations:** 1 AGEIS Université Grenoble Alpes La Tronche France; 2 Human Movement Research Laboratory Department of Physical Education. School of Sciences São Paulo State University Bauru Brazil; 3 Department of Computer Science and Applied Mathematics Weizmann Institute of Science Rehovot Israel; 4 Institut Universitaire de France Paris France

**Keywords:** obesity, obstacle crossing, gait, systematic review, overweight, weight, obstacle, walking, balance, fall, risk, prevention, mobility

## Abstract

**Background:**

Overweight and obesity are significant global health concerns that involve deficits in gait and balance that affect daily activities. Although much is reported about the effect of overweight and obesity on gait during unobstructed walking, not much is known about how overweight and obesity could impact gait under more challenging conditions, such as environments with obstacles.

**Objective:**

The aim of this study is to systematically review and synthesize the available data regarding the effects of overweight and obesity on obstacle crossing during walking.

**Methods:**

This review will follow the PRISMA (Preferred Reporting Items for Systematic Reviews and Meta-analyses) guidelines. PubMed, Web of Science, Scopus, and SPORTDiscus will be systematically searched with no limitations on publication date. Only full-text English-language articles published in a peer-reviewed journal will be included. Included articles must have compared obstacle crossing during walking in individuals with overweight or obesity to individuals of normal body weight. A total of 2 independent reviewers will select the articles and extract the following 4 sets of data: (1) study characteristics, (2) sample description, (3) obstacle crossing task protocol, and (4) main results obtained. If a considerable number of homogeneous papers are included, a meta-analysis will be conducted. A preliminary search was conducted in November 2021.

**Results:**

The results will include the article selection flowchart as well as tables and figures synthesizing the extracted data on the effects of overweight and obesity on obstacle crossing during walking. The preliminary search identified 73 original records, of which 5 articles met the inclusion criteria.

**Conclusions:**

This review will present researchers and clinicians with an overview of published studies that have compared the performance of obstacle crossing for individuals with overweight and obesity to those of normal body weight. Gaining insight into the control strategies adopted by individuals with overweight and obesity is critical for safe and successful obstacle crossing in this population. We therefore believe that our findings could be useful for identifying people at risk of falls and developing and implementing fall prevention programs for individuals with overweight and obesity.

**Trial Registration:**

PROSPERO CRD42021269949; https://tinyurl.com/3yrwccu4

**International Registered Report Identifier (IRRID):**

DERR1-10.2196/36234

## Introduction

Overweight and obesity are defined as abnormal or excessive fat accumulation that presents a risk to health. According to the US Centers for Disease Control and Prevention, individuals with a BMI between 25 kg/m^2^ and 29.9 kg/m^2^ are considered overweight, and those with a BMI over 30 kg/m^2^ are considered obese. Overweight and obesity have become a major public health issue, and the incidence of the condition is increasing at an alarming rate worldwide [1]. Recent data from the World Health Organization’s Global Health Estimates report that the worldwide prevalence of obesity nearly tripled between 1975 and 2016 and that in 2016, 39% of the world’s adult population was overweight and 13% was obese [2]. Overweight and obesity are associated with, among other things, impaired postural balance and gait limitations. Interestingly, biomechanical alterations imposed by the additional loading of the locomotor system have been analyzed and reported in recent reviews (eg, [3-5]). For instance, Molina‐Garcia et al [4] recently reported strong evidence that suggests that gait patterns of children and adolescents with overweight and obesity are characterized by greater pelvis transversal plane motion, higher hip internal rotation and flexion, extension and abduction moments and power generation and absorption, greater knee abduction and adduction motion, and higher knee abduction and adduction moments and power generation and absorption compared with normal-weight counterparts. These biomechanical alterations observed in individuals with overweight and obesity during locomotor tasks further have been reported to significantly increase the risk of musculoskeletal disorders, especially in lumbar, hip, and knee regions (see [3,6] for reviews), the risk of injury while performing activities of daily living (ADL), functional limitations (eg, see [6-8] for recent reviews), and the risk of falls and multiple falls (eg, see [9] for a recent systematic review). We can first mention the systematic review and meta-analysis by Backholer et al [7] that demonstrated a graded increase in the risk of ADL limitations from normal weight (BMI 18.5 kg/m^2^ to 24.9 kg/m^2^) to overweight (BMI 25 kg/m^2^ to 29.9 kg/m^2^), obesity class I (BMI 30 kg/m^2^ to 34.9 kg/m^2^), and obesity class II+ (BMI >35 kg/m^2^). Additionally, the systematic review and meta-analysis by Neri et al [9] showed that people with obesity over 60 years have a 16% increase in the risk of falls compared to older adults of normal weight.

At this point, however, although much is reported about the deleterious effect of overweight and obesity on gait during unobstructed walking (eg, see [10-16] for recent works published in 2021), not much is known about how overweight and obesity could impact gait under more challenging conditions, such as an environment with obstacles (eg, [12,17-20]). Interestingly, indeed, crossing an obstacle while walking is a challenging task, reflecting a higher risk of loss of balance, trips, and falls [21-26]. Actually, trips over an obstacle are one of the main causes (accounting for up to 53%) of falls during walking in healthy older adults [27]. It has been suggested that these observations could stem from the increased neuromuscular demand of obstacle crossing during walking in comparison to unobstructed walking [23,28,29]. Accordingly, considering the functional limitations imposed by the additional loading of the locomotor system in individuals with overweight or obesity, it is of particular interest to evaluate whether and how overweight and obesity impair obstacle crossing during walking.

To the best of our knowledge, the available evidence on the effect of overweight and obesity on obstacle crossing during walking has not yet been systematically reviewed, and by identifying and synthesizing the current evidence on the topic, we can provide new insights on the potential influence of overweight and obesity on walking in an environment with obstacles. This work was therefore designed to address this issue. Our aim was to conduct a systematic review of published studies that have compared gait parameters related to the performance of the obstacle crossing task in individuals with overweight or obesity and those of normal body weight.

## Methods

### Guidelines and Registration

The PRISMA (Preferred Reporting Items for Systematic Reviews and Meta-analyses) [30] statement and recommendations and Cochrane Handbook for Systematic Reviews guidelines [31] will be used for this systematic review to identify relevant studies. We will systematically review published studies that have described and compared obstacle crossing during walking in individuals with overweight or obesity and individuals of normal body weight. The protocol for this systematic review has recently been registered in PROSPERO (the International Prospective Register of Systematic Reviews; CRD42021269949).

### Eligibility Criteria

Only full-text, peer-reviewed, scientific original articles published in English will be included in this review. Case reports, abstracts, editorials, letters to the editor, case studies, books, chapters, reviews, meta-analyses, and other grey literature materials (government reports, policy statements and conference proceedings, preprints, theses, and dissertations) will be not included. We will use the eligibility criteria below for study selection.

#### Population

Studies on individuals with overweight or obesity (defined as a BMI ≥25 kg/m^2^) may be included. Studies using animal models will be excluded.

#### Intervention

Studies using the following intervention were considered: obstacle crossing during walking.

#### Comparator

Studies where individuals with overweight or obesity (defined as BMI ≥25 kg/m^2^) and those of normal body weight (BMI 18 kg/m^2^ to 24.9 kg/m^2^) were compared will be considered.

#### Outcomes

Studies including spatial-temporal, angular, kinetic, and muscle activation outcomes related to the performance of the obstacle crossing task will be considered.

### Data Sources and Search Strategy

A total of 4 electronic databases, PubMed, Web of Science, Scopus, and SPORTDiscus, will be searched systematically from inception onwards to identify studies satisfying the search criteria. The search strategy includes a combination of the following keywords related to (1) the population (individuals with overweight or obesity) and (2) the intervention (obstacle crossing), using the Boolean operators “AND” and “OR” and, if applicable, Medical Subject Headings terms: “obes*” OR “overweight” OR “over‐weight” OR “adipos*” OR “body mass index” OR “BMI”. The second category specifies the intervention task comprising a term related to obstacle crossing, such as the following: “obstacle crossing” or “obstacle negotiation” or “obstacle avoidance” or “obstructed walking”.

### Study Selection

A total of 2 independent reviewers (MC and TP) will screen the titles, abstracts, and keywords of each article retrieved from the electronic database searches. Inclusion is based on the previously mentioned selection criteria. The same 2 independent reviewers (MC and TP) will screen the full-text articles for inclusion. A third reviewer (NV) will be consulted in cases of disagreement between the 2 reviewers.

### Data Extraction

After completion of the screening process, the same 2 independent reviewers (MC and TP) will extract the following 4 categories of data from each included article: (1) study characteristics, (2) sample description, (3) obstacle crossing task protocol, and (4) main results. Means and standard deviations or medians associated with interquartile ranges or first and third quartiles will be extracted. In the case of missing or erroneous data, the study authors will be contacted for further information. Both independent reviewers will compare the data for consistency. Any disagreement between the 2 independent reviewers will be resolved by consensus or discussion with a third reviewer (NV).

### Data Synthesis and Analysis

This systematic review is specifically designed to present an overview of the existing literature on the effect of overweight and obesity on obstacle crossing during walking. We will therefore systematically review published articles that have described and compared spatial-temporal, angular, kinetic, and muscle activation outcomes related to the performance of an obstacle crossing task for individuals with overweight or obesity and those of normal body weight. We will also report the magnitude of the potential differences (as percentages) between individuals with overweight or obesity and those of normal body weight. In the case of a considerable amount of nonheterogeneous studies that meet the eligibility criteria being included, we will verify the possibility of conducting a meta-analysis using specific packages in R (R Foundation for Statistical Computing). The risk of bias of the included studies will be assessed using the Cochrane Collaboration’s tool [32] and a specific tool created for a review with a similar scope [33]. Specifically, 2 independent reviewers (MC and TP) will examine the full texts regarding random sequence generation, allocation concealment, blinding of participants and personnel, blinding of the outcome assessment, incomplete data, selective reporting, and other biases (ie, not covered by other criteria). Each criterion will be classified as low-risk (no bias or minimal effects on results), unclear risk (not specified or raises some doubt about the results), and high-risk (may alter the results) [32]. Considering the specific grid created by Galna et al [33], questions will be related to the internal validity, external validity, and reproducibility of the methods related to obstacle crossing. Each question on the quality assessment tool will be scored. A score of 1 will indicate that the study meets the assessment criterion, while a score of 0 will indicate that the assessment criterion is not met. A score of 0.5 will indicate a lack of information or a lack of clarity in the corresponding items. Any disagreement between the 2 reviewers will be resolved by consensus or discussion with a third reviewer (NV).

### Ethical Considerations

As this review is limited to publicly available materials, it does not require ethical approval. Results will be shared with the scientific community and the general public.

## Results

The search strategy described above was completed in November 2021 and led to 133 records; after removing duplicates, 73 records were identified. After screening titles and abstracts, 5 full texts were reviewed, and all of these articles were included in the review. Data extraction and synthesis are currently ongoing. In line with PRISMA guidelines [30], the number of citations reviewed at each stage of the systematic review will be summarized in a flow chart. The risk of bias of the included studies will be described [32]. Tables and figures summarizing the extracted data will be produced. The dissemination of study results to an international audience through publication in a peer-reviewed journal is expected at the end of 2022.

## Discussion

We hypothesize that evidence will show how overweight and obesity negatively affect gait during obstacle crossing. Presumably, individuals with overweight and obesity, due to individual constraints imposed by the obesity on one’s gait, would have a higher risk of slips, trips, and falls during obstacle crossing. [27] Obstacle crossing represents an everyday life situation that has been shown to challenge balance and increase the risk of falls [21-26]. Obstacle crossing is therefore an important task to consider in individuals with overweight and obesity [34] and may be useful in identifying those at risk of falls, preventing falls, and reducing the risk of fall-related injuries. However, it is surprising that systematically analyzed data on the effects of overweight and obesity on obstacle crossing during walking are rather scarce. In fact, based on our initial screening process, only a limited number of published articles successfully met all of the eligibility criteria (n=5; [12,17-20]). A total of 2 articles focused on children [17,18], 2 on adults [12,20], and 1 on postmenopausal women [19]. The number of participants with overweight or obesity included in these 5 studies ranged from 12 [17,18] to 54 [20]. Of these studies, 4 [12,17,18,20] assessed the effects of 3 fixed obstacle heights (low, medium, and high obstacles, measuring from 4 cm to 16 cm in height) on obstacle crossing; 1 study [19] used an obstacle height condition that was based on the length of the participants’ lower limbs (30%). The lower-limb kinematics of participants with overweight or obesity and those with a normal BMI as they crossed over obstacles were reported in all studies [12,17-20]; 1 study further collected kinetic data that were time-synchronized with kinematic data [18]. A preliminary and very synthetic analysis tends to show that overweight and obesity impair gait patterns during obstacle crossing in children [17,18], adults [12,20], and postmenopausal women [19].

To the best of our knowledge, this will be the first systematic review to date identifying and synthesizing the available evidence on the effect of overweight and obesity on obstacle crossing during walking. The systematic review was registered in PROSPERO (CRD42021269949). This systematic review will follow the PRISMA statement and recommendations [30] and the Cochrane Handbook for Systematic Reviews guidelines [31] to develop high-quality research questions, capture relevant studies, and critically appraise the relevant studies. A total of 2 independent reviewers will screen titles, abstracts, keywords, and full-text articles and rate the quality of these studies and the risk of bias. A synthesis will then be provided with the information presented in the main text, tables, and figures to summarize the characteristics and main findings of the included studies. On completion, the results of this systematic review will be presented at national and international conferences and submitted for publication in a peer-reviewed scientific journal.

We anticipate that the results of this systematic review will help researchers and health professionals increase the quality of care for people with overweight and obesity. We believe that gaining insight into the mechanisms and strategies adopted by individuals with overweight and obesity is critical for safe and successful obstacle crossing in this population. Along these lines, our findings could be useful for identifying individuals with overweight or obesity at risk of falls and developing and implementing tailored fall prevention programs for these individuals.

## Abbreviations

ADL: activities of daily living
PRISMA: Preferred Reporting Items for Systematic Reviews and Meta-analyses
PROSPERO: International Prospective Register of Systematic Reviews

## Data Availability

Data sharing is not applicable to this article as no data sets were generated or analyzed during the current study.
